# BCG vaccination policy, natural boosting and pediatric brain and CNS tumor incidences

**DOI:** 10.3389/fimmu.2023.1174006

**Published:** 2023-06-13

**Authors:** Samer Singh, Amita Diwakar, Rakesh K. Singh

**Affiliations:** ^1^ Centre of Experimental Medicine & Surgery, Institute of Medical Sciences, Banaras Hindu University, Varanasi, India; ^2^ Department of Obstetrics and Gynecology, Institute of Medical Sciences, Banaras Hindu University, Varanasi, India; ^3^ Department of Biochemistry, Institute of Science, Banaras Hindu University, Varanasi, India

**Keywords:** cancer discovery, pediatric cancer, brain and CNS cancer, childhood vaccinations, BCG vaccine, trained immunity, cancer prevention, *Mycobacterium* spp.

## Abstract

Bacille Calmette-Guérin (BCG) vaccination supposedly imparts and augments “trained immunity” that cross-protects against multiple unrelated pathogens and enhances general immune surveillance. Gradual reductions in tuberculosis burden over the last 3–5 decades have resulted in the withdrawal of BCG vaccination mandates from developed industrialized countries while reducing to a single neonatal shot in the rest. Concurrently, a steady increase in early childhood Brain and CNS (BCNS) tumors has occurred. Though immunological causes of pediatric BCNS cancer are suspected, the identification of a causal protective variable with intervention potential has remained elusive. An examination of the countries with contrasting vaccination policies indicates significantly lower BCNS cancer incidence in 0–4-year-olds (per hundredthousand) of countries following neonatal BCG inoculations (n=146) *vs*. non-BCG countries (n=33) [Mean: 1.26 *vs*. 2.64; Median: 0.985 *vs*. 2.8; IQR: 0.31–2.0 *vs*. 2.4–3.2; P=<0.0001 (two-tailed)]. Remarkably, natural *Mycobacterium* spp. reexposure likelihood is negatively correlated with BCNS cancer incidence in 0-4-year-olds of all affected countries [*r*(154): −0.6085, *P*=<0.0001]. Seemingly, neonatal BCG vaccination and natural “boosting” are associated with a 15–20-fold lower BCNS cancer incidence. In this opinion article, we attempt to synthesize existing evidence implying the immunological basis of early childhood BCNS cancer incidence and briefly indicate possible causes that could have precluded objective analysis of the existing data in the past. We draw the attention of the stakeholders to consider the comprehensive evaluation of immune training as a potential protective variable through well-designed controlled clinical trials or registry-based studies as feasible for its potential applications in reducing childhood BCNS cancer incidence.

## Introduction

Bacille Calmette-Guérin (BCG), a derivative of *Mycobacterium bovis* (a member of *M. tuberculosis* complex*)*, is the most widely used early childhood vaccine that is in use for over 100 years for protecting against tuberculosis (TB) (1). It offers the greatest protection against miliary TB and tuberculous meningitis and to a lesser extent against pulmonary TB (2). However, the non-specific protection offered by BCG against other common pathogens, sepsis, and unrelated conditions has been associated with up to a 50% reduction in early childhood mortality rate in different studies (3–7). BCG is supposed to provide this protection through the induction of granulopoiesis, the activation of heterologous T-cell immunity, and enhanced non-specific innate immunity committed through epigenetic and metabolic reprogramming (8–15). Mechanistically, the inoculation is supposed to bring about functional reprogramming of the cells of innate immune response (*e.g*., Monocytes, macrophages, NK cells, etc.) that leads to better immune surveillance and response (9–16). In the case of children growing up in ‘hygienic’ conditions, it has been hypothesized to provide the necessary immune stimulus required for “normal” immune system development that may reduce the incidence of common childhood cancers and immune conditions, including autoimmune diseases (17–21). The ability of BCG to potentiate cell-mediated response, which is believed to be important for cancer, has led to its evaluation for preventive and therapeutic potential in many observational studies and clinical trials (15, 18–20, 22–28). However, except in the case of bladder cancer, its preventive and therapeutic potential remains debated and largely uncertain due to the lack of comprehensive and consistent data along with a theoretical framework that could at least attempt to explain different conflicting observations (15, 19, 20, 22). The evidence generated so far seems to be overwhelmingly negating the potential of BCG in protecting against cancers. We envision this apparent uncertainty about the potential benefits of BCG vaccination and conflicting outcomes in different studies to be resulting from the inappropriate generalization of the potency of different BCG vaccines in activating the innate immune system (29, 30), omissions about the requirement of boosters (31, 32), longevity of conferred non-specific “trained immunity” that seldom lasts a few years in the absence of a booster (31–33), treating all cancers as a homogeneous lot disregarding their origin (*e.g*., embryonal, mutational: sporadic, germline, primary, secondary, *etc.*) and associated inherent differences, non-consideration of the immune status of subjects and their exposure to a specific intervention and risk factors, *etc.* in different studies reported in (10, 15, 17–20, 22).

## Early childhood cancers and “Hygiene”

Early childhood cancers are hypothesized to result from aberrant embryonic remnants that could have been otherwise eliminated during ‘normal’ development had the appropriate immune stimulus and training been available in the form of common pathogens and microbes to which we have been exposed during evolution (17–20, 22, 28). Based on the observation of children brought up in supposedly more hygienic conditions, a critical role for natural exposure to pathogens for immune reprogramming and maturation has been proposed (17–20, 34–36). ‘Hygienic’ conditions have been associated with increased allergy and atopic conditions, susceptibility to life-threatening infections, and sometimes even with the differential incidence rates of cancers (9, 11, 17, 19–22). However, for the lack of consistent evidence pinpointing a preventive/protective agent or intervention that may have a direct causal relationship, ambivalence about their actual potential in cancer incidence prevention has remained (15, 17, 19, 22, 28).

## Changing incidence of early childhood brain and central nervous system cancer and potential risk factors

Brain and other central nervous system (BCNS) cancer remains the second most frequently occurring childhood cancer and the most frequent cause of cancer mortality (37). The updated estimates for all cancers and ages periodically produced by the International Agency for Research on Cancer, WHO as a part of the GLOBOCAN project (https://gco.iarc.fr/today/home) are a comprehensive source of cancer incidence estimates and related information. Incidentally, the industrialized western world with high living standards and hygiene has higher incidence rates of both early childhood and later-age BCNS cancer (37). Most of the countries for which estimates are available have been steadily registering an annual increase in childhood BCNS cancer incidence for the last 3-5 decades (37). The improvement in TB prevalence and incidence resulting from improved hygiene and living standards globally had also seen a concomitant reversal of BCG mandates, and changes in BCG booster schedules, or altogether scrapping in several countries (38, 39). For the majority of countries/territories with data, the estimated annual % change in total BCNS cancer incidence has been positive for recent decades (**Supplementary Figure 1**) (37). The childhood BCNS cancer incidence is highest in 0-4-Year-olds (0-4Y-Old or younger than 5 years) and decreases in older 5-14Y-old children. GLOBOCAN study estimates BCNS cancer incidence and associated mortality among 0-4Y-olds during the year 2020 to be 9252 and 4256, respectively, which accounted for about 16% of incidence and 20% of associated deaths from all cancer types combined (37). Despite, advances in diagnostics and therapeutics, the preventive vaccines and causative risk factors for the majority of early childhood BCNS cancers remain unidentified and uncharacterized (40–43). Though previously the occurrence of malignant and embryonic origin BCNS tumors in children <5 years has been variously associated (positively as well as negatively) with risk factors like socioeconomic position (41, 42, 44), allergy (42, 45, 46), infections or exposure to pathogens early in life (47, 48), human developmental index (HDI), etc. (41–48), the identification of potential protective risk factors with an intervention potential (*i.e.*, employable or actionable) to reduce the incidence of BCNS cancers has so far remained an unfulfilled aspiration.

## BCNS cancer origin and BCG vaccination

Compared to BCNS cancers arising in any other age group, the early childhood incidences are predominantly of embryonal origin and malignant, and their incidence rates decrease with age (40–43). The scientific community may be aware of the fact that although globally BCNS cancer incidence rates pick up again around 20 years of age and keep going up with age (37, 40, 41), they are overwhelmingly not of the same origin (up to 10-fold) (42, 43). This offers an opportunity to evaluate any potential role of immune modulation and maturation, more specifically that of BCG vaccination, the most widely given vaccine, on the incidence of childhood BCNS cancer, if any. We surmise that if early childhood BCG vaccination-induced/primed non-specific trained immunity could play any role in cancer incidence arising from abnormal immune system development and maturation, their effect would be more discernible in early childhood cancers, which allegedly arise from embryonic remnants that could not be eliminated during early development for the lack of appropriate and timely immune system stimulation (priming and maturation). The policy on BCG vaccination varies by country (**Table 1**) (39). Currently, the majority of middle- to low-income countries follow the BCG vaccination policy for newborns, while non-BCG mandating countries (No-BCG) are mostly affluent high-income countries (51) with very high (vh-) to high (h-) HDI – a composite measure of “a long and healthy life, access to knowledge and a decent standard of living” (49). The existence of disparities in BCG vaccination policies (39) and TB incidence rates (*i.e.*, *M. tuberculosis* complex exposure possibility) across countries (50) provides a unique opportunity to test the hypothesis that BCG vaccination and/or “boosting” may be potentially protective (alternatively non-protective) in early childhood BCNS cancer.

**Table 1:** Early Childhood Brain and other Central Nervous System (BCNS) cancer incidence in different HDI group countries with varying tuberculosis (TB) incidence rates (potential exposure) and correlation analysis.

BCNS Cancer Incidence (BCNS Can. Inc.) in 0-4Y-olds (0-4Y) and TB incidence (TB Inc.) rates per 100,000 (100k) for Countries, 2020															
Very High ^a^HDI (vh-HDI) (49)			High ^a^HDI (h-HDI) (49)				Medium ^a^HDI (m-HDI) (49)					Low ^a^HDI (l-HDI) (49)			
Country/ Territory [with ‘No BCG’ for all policy (NB) (39)	*^b^* BCNS Can. Inc. per 100k (37)	*^c^* TB Inc. per 100k (50)	Country/Territory [with ‘No BCG’ for all policy (NB) (39)	*^b^* BCNS Can. Inc. per 100k (37)		*^c^* TB Inc. per 100k (50)	Country/ Territory		*^b^* BCNS Can. Inc. per 100k (37)		*^c^* TB Inc. per 100k (50)	Country/ Territory		*^b^* BCNS Can. Inc. per 100k (37)	*^c^* TB Inc. per 100k (50)
United States (NB)	4.3	2.3	Suriname (NB)	3.8		29	Cape Verde		3.9		39	Yemen		1.5	48
Greece (NB)	4.2	4.2	Lebanon (NB)	2.3		13	Syria		2.2		18	Afghanistan		1	189
Canada (NB)	4.1	5.4	North Macedonia	3.6		12	Venezuela		2		44	*North Korea		0.97	513
Israel (NB)	4	2.1	Moldova	3.5		78	Iraq		2		26	Nigeria		0.83	219
Slovenia (NB)	3.9	4.1	Albania	3		16	Morocco		1.9		96	Pakistan		0.65	255
Slovakia (NB)	3.9	3.2	Armenia	2.9		25	El Salvador		1.7		54	*Somalia		0.64	254
Australia (NB)	3.2	7.2	Mexico	2.7		23	Nicaragua		1.7		42	Ethiopia		0.61	129
Germany (NB)	3.2	5.3	Palestine	2.7		0.65	Kyrgyzstan		1.3		108	Malawi		0.55	138
Portugal (NB)	3.2	16	Ukraine	2.6		74	Honduras		1.3		30	Uganda		0.51	199
United Kingdom (NB)	3	7	Jordan	2.5		4.6	Laos		1.3		149	Mozambique		0.43	361
Italy (NB)	3	4.2	Algeria	2.4		58	Cambodia		1.3		280	Rwanda		0.42	57
Ireland (NB)	2.9	5.3	Cuba	2.3		6.3	India		1.1		204	South Sudan		0.41	227
Netherlands (NB)	2.9	4.1	Egypt	2.3		11	Tajikistan		0.96		80	Eritrea		0.4	80
Austria (NB)	2.9	4.9	Iran	2.2		12	Ghana		0.89		140	Lesotho		0.39	592
Belgium (NB)	2.8	7.7	Peru	2.2		117	Zimbabwe		0.81		181	Mali		0.36	51
Spain (NB)	2.8	7.2	Brazil	2.2		45	Zambia		0.75		319	Senegal		0.34	115
Norway (NB)	2.7	3.1	Ecuador	2.2		44	Eswatini		0.7		342	Burundi		0.34	103
New Zealand (NB)	2.7	7.2	Colombia	2		34	Kenya		0.7		250	Chad		0.34	141
France (NB)	2.7	8.3	Libya	1.8		59	Bolivia		0.67		103	Guinea-Bissau		0.33	361
Czechia (NB)	2.7	3.9	China	1.7		57	Timor		0.56		473	Niger		0.31	82
Denmark (NB)	2.6	4.1	Jamaica	1.7		3.1	Myanmar		0.55		311	Sudan		0.3	62
Sweden (NB)	2.5	3.5	Tunisia	1.6		36	Guatemala		0.53		25	Sierra Leone		0.26	292
Switzerland (NB)	2.4	4.7	Bosnia and Herzegovina	1.5		26	Mauritania		0.43		85	Madagascar		0.17	233
*Puerto Rico (NB)	2.4	0.95	Dominican Republic	1.4		41	Bangladesh		0.32		221	Burkina Faso		0.17	46
Finland (NB)	2.3	3.6	Philippines	1.3		533	Namibia		0.3		451	Togo		0.16	35
Trinidad and Tobago (NB)	2.3	17	Turkmenistan	1.2		46	Nepal		0.3		230	Democratic Republic of Congo		0.16	319
Cyprus (NB)	1.5	3.2	Uzbekistan	1.2		65	Angola		0.26		329	Liberia		0.14	308
Iceland (NB)	0	3.8	Paraguay	1.1		45	Cameroon		0.22		171	Guinea		0.14	175
Luxembourg (NB)	0	5.8	Vietnam	1.1		171	Cote d'Ivoire		0.1		132	Central African Republic		0.14	540
Malta (NB)	0	31	Sri Lanka	1		63	Belize		0		27	Tanzania		0.08	222
Bahamas (NB)	0	8.8	Azerbaijan	0.85		58	Bhutan		0		164	Haiti		0	164
Montenegro	5.4	15	Indonesia	0.79		301	Sao Tome and Principe		0		115	Benin		0	54
Estonia	4.3	10	Botswana	0.74		230	Vanuatu		0		37	Djibouti		0	220
Croatia	3.8	5	South Africa	0.68		562	Equatorial Guinea		0		275	Gambia		0	153
Latvia	3.5	20	Mongolia	0.53		428	Congo		0		372				
Belarus	3.5	28	Gabon	0.31		517	Solomon Islands		0		58				
Lithuania	3.4	28	Barbados	0		2.5	Comoros		0		35				
Poland	3.3	9.4	Fiji	0		66	Papua New Guinea		0		418				
Hungary	3.3	4.5	Maldives	0		37									
Serbia	3.3	14	Saint Lucia	0		2.2									
Russia	3.1	48	Guyana	0		78									
Turkey	3	16	Samoa	0		7									
Uruguay	3	32													
Chile	2.7	14													
Malaysia	2.7	90													
Japan	2.5	12													
South Korea	2.3	48													
Costa Rica	2.3	9.7													
Bulgaria	2.2	19													
Georgia	2.2	69													
United Arab Emirates	2	0.77													
Oman	2	7.9													
Singapore	1.9	46													
Argentina	1.6	28													
Romania	1.6	43													
Mauritius	1.6	12													
Thailand	1.6	144													
Qatar	1.5	36													
Saudi Arabia	1.4	7.8													
Kazakhstan	1.4	66													
Kuwait	1	18													
Panama	1	33													
Bahrain	0.93	15													
*Guam	0	39													
Brunei	0	82													
											
***Average (vh-HDI)***	2.47	19.53	***Average (h-HDI)***	1.62		96.10	***Average (m-HDI)***		0.81		169.32	***Average (l-HDI)***		0.38	204.03
***STDEV***	1.18	25.09	***STDEV***	1.05		147.18	***STDEV***		0.83		131.61	***STDEV***		0.32	141.47
											
	**AVERAGE (ALL COUNTRIES*)***													**1.52**	**104.34**
	**STDEV (ALL COUNTRIES)**													**1.27**	**134.92**
**CORRELATION ANALYSIS (BCNS Cancer Inc. in 0-4Y-olds and TB Inc. per 100k)**															
**Countries**	**ALL**		***HDI SUBGROUP COUNTRIES***										***Note:*** *1.* The values shown are rounded off to the indicated decimal places; STDEV: standard deviation. 2. The exclusion of zero incidence countries from “Total” analyzed, further improves the association [*i.e*., ***r*(154): −0.6085, P = <0.0001**]. Exclusion of outliers in No-BCG and BCG Countries, *i.e.*, Incidence rate ‘0’ and Maximum ‘5.4’, respectively or only consideration of countries with BCNS Inc.: >0 to 4.3, did not adversely affect the correlation for groups ‘All countries’ [***r*(153): −0.6151; *P =* <0.0001**] or ‘BCG Countries’ [***r*(124): −0.5679; *P =* < 0.0001**].		
			***Very High HDI***		***High-HDI***			***Medium-HDI***		***Low-HDI***					
**Total: Affected + Not Affected** *(BCNS Inc.: 0–5.4)*	***r(*179): −0.4973;** ***P =* < 0.0001 **		***r*(65): −0.2990;** ***P =* 0.0155 **		***r*(42): −0.3446;** ***P =* 0.0254 **			***r*(38): −0.3840; *P =* 0.0173 **		*r*(34): 0.0271; *P =* 0.8789					
**Total: AFFECTED ONLY** (BCNS Inc.: >0 – 5.4)	***r(*154): −0.6085;** ***P =* < 0.0001 **		***r*(59): −0.3212;** ***P =* 0.0131 **		***r*(36): −0.5975;** ***P =* 0.0001 **			***r*(29): −0.5468; *P =* 0.0021 **		*r*(30): -0.0413; *P =* 0.8283					
					
***No-BCG (NB) Only***	r(33): −0.2179; *P* ***=*** 0.2232		***r*(31): −0.3903;** ***P =* 0.0299 **		**N.D.**			-		–					
***BCG: Affected + Not affected***	***r*(146): −0.4581;** ***P =* < 0.0001 **		***r*(34): −0.3257;** ***P =* 0.0601 **		***r*(40): −0.3339;** ***P =* 0.0352 **			***r*(38): −0.3840; *P =* 0.0173 **		*r*(34): 0.0271; *P =* 0.8789					
***BCG: AFFECTED ONLY***	***r*(125):** −**0.5595;** ***P =* < 0.0001 **		*r*(32): −0.2453; *P =* 0.1759		***r*(34): −0.6072;** ***P =* 0.0001 **			***r*(29): −0.5468; *P =* 0.0021 **		*r*(30): -0.0413; *P =* 0.8283					

**^a^Human Development Index (HDI) 2020:** A composite index used by United Nations Development Program (UNDP) to indicate overall human development in three basic dimensions, i.e., “a long healthy life, knowledge base and a decent standard of living” (49). Based on HDI of countries they are stratified into very high (>0.8), high (0.7-0.799), medium (0.550–0.699), and low (<0.550). *Not listed by UNDP. Some countries were shown as vh- and h-HDI by UNDP in 2020 but not by IACR (37) were considered as such for correlation analysis.

**^b^(37).** **Age-standardized rate (ASR) estimates for the incidence of BCNS cancer in 0-4Y-old (2020) children** are from the “GLOBOCAN 2020”, International Agency for Research on Cancer (IARC), World Health Organization. The details of research methodology employed by GLOBOCAN 2020 study for estimating the global cancer incidences are available at the Global Cancer Observatory (GCO) website [https://gco.iarc.fr/today/home; Last accessed 15 December 2022].

**^c^(50). The estimates of TB incidence for countries in 2020** **from Global Tuberculosis Programme:** WHO TB burden estimates. Available at https://www.who.int/teams/global-tuberculosis-programme/data (50) [Last accessed 05 Feb 2023].

In Correlation analysis, the bold values are significant ones while the normal values are the non-significant ones.

## BCG countries frequently have lower incidence of early childhood BCNS cancer

**Figure 1:**
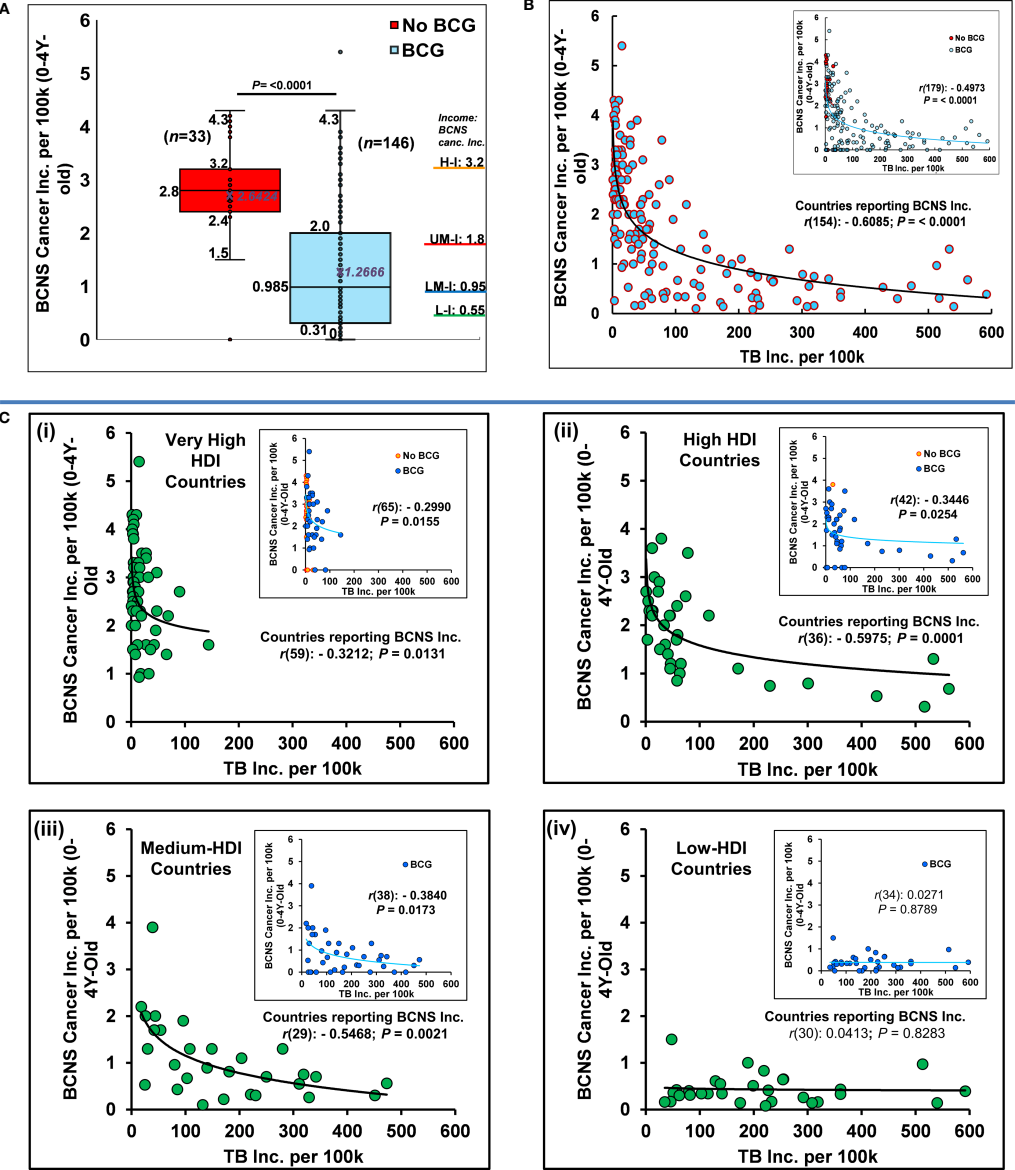
BCG vaccination and *M. tuberculosis* complex exposure negatively associated with BCNS cancer incidence in 0-4Y-old children. **(A)** Globally, the distribution of age-standardized BCNS cancer incidence rates (ASR) among countries (n=179) are shown as Box and whisker plot. BCG-vaccinated children had significantly reduced incidence (t-test: two-tailed, equal/unequal variance) than the non-vaccinated (No BCG). The average incidence rate seems directly proportional to income levels [H-I: High ($13,205 or more); UM-I: Upper Middle (between $4,256 and $13,205); LM-I: Lower Middle ($1,086 and $4,255); L-I: Low income (≤$1,086) as defined by World Bank, 2023 (51) (For CI refer to (37)]. **(B)** The probability of natural boosting (exposure to the *M. tuberculosis* complex) is strongly associated with lower BCNS cancer incidence in the affected countries/territories. **(C)** BCNS cancer incidence in countries belonging to different HDI subgroups: The distribution of BCNS cancer incidence with TB incidence rates among very high HDI [panel C(i)], high HDI [panel C(ii)], medium HDI [panel C(iii)], and low HDI [panel C(iv)] countries indicates a significant negative correlation between BCNS cancer incidence and the supposed probability of *M. tuberculosis* complex exposure among countries except in the group of low-HDI countries (also refer to **Table 1** for subgroup analysis). Inset has all countries color-coded for BCG and No BCG groups. The trend lines are drawn to guide the eye. *Note* the presence of outliers in the No-BCG and BCG groups. Most of the high BCNS incidence-displaying countries of the BCG group, including the outlier, are clustered in low background *M. tuberculosis* complex exposure regions with TB incidence rates of < 100 per 100k **(B, C)** for the year 2020.

Globally, the distribution of updated age-standardized incidence rates (ASR) of BCNS cancer for the recent year 2020 (37) varies widely from 0 to 5.4 per 100,000 (100k) in 0-4Y-olds (**Table 1**). Nevertheless, the widely mandated BCG vaccination seems associated with reduced childhood BCNS cancer incidence in 0-4Y-olds (**Figure 1A**). The countries of Europe and North America with no neonatal BCG vaccination policy in place (‘No-BCG’ or ‘NB’) are some of the worst affected countries, while incidence rates in countries with BCG vaccination in place (BCG countries) do have some similar high incidence rate countries, but the majority seem to cluster towards lower incidence rates. The incidence in No-BCG and BCG countries significantly differ (Mean: 2.64 *vs* 1.26; Median: 2.8 *vs* 0.985; IQR; 2.4–3.2 *vs* 0.31–2.0; *P* = <0.0001 (two-tailed)). The high to low incidence rates of BCNS cancer among countries also display an interesting association with countries’ income brackets (51). The higher income brackets have higher average incidence rates than the lower income brackets (**Figure 1A**: right side Y axis). The previous observations made in small-scale studies performed in different countries that identified the socioeconomic position (SEP) of families as a potential risk factor positively associated with childhood BCNS cancer incidence (52, 53), seem to be applicable globally. However, the occurrence of high incidence rates (ASR) in certain BCG vaccinating countries of Europe [notably, Montenegro (5.4), Estonia (4.3), Croatia (4.3), North Macedonia (3.6), Republic of Moldova (3.5), Lithuania (3.4), Serbia, Poland (3.3)] along with wide variation in incidence rates among BCG vaccinating countries 0 – 5.4, casts doubt on the ability of single BCG shots alone to protect young children from BCNS cancers, as could have been the case in some of the past studies reported in (15, 19, 20, 22) that failed to find an association between BCG vaccination and cancer incidence.

## Globally, higher natural “boosting” potential negatively associated with pediatric BCNS cancer incidence in countries

A missing explanatory variable for the observed reduced BCNS cancer incidence in BCG countries could be the ‘boosting’ stimulus arising from natural exposure to boosting events (also priming for No-BCG countries) in the form of *M. tuberculosis* complex exposure specific to populations. It may be pertinent here to reiterate for the scientists from other backgrounds that *M. tuberculosis* complex exposure does not invariably cause TB but rather only sustained long-term exposure of susceptible individuals, which constitutes a small fraction of the population – variously estimated to be <1-5%, is supposed to result in clinical TB (33, 54). Assuming the BCG vaccination could be playing a role in the BCNS cancer incidence in young children, the higher incidence in some countries could be envisioned to result from the inability of a single BCG inoculation to sufficiently activate the immune system in the absence of proper, timely boosting. Revaccination has been indicated to enhance the non-specific protective effects of BCG in early childhood (3, 55, 56). The neonates in countries with higher TB prevalence or incidence rates (50) could be more frequently exposed to natural boosting events than those born in low TB incidence countries.

Surprisingly, when we look at the covariation of BCNS cancer incidence rates (37) with countries’ TB incidence rates (50) they are found to be significantly negatively correlated even when completely disregarding their BCG vaccination policy (**Table 1**). This correlation further improves on consideration of the countries reporting childhood BCNS cancer incidence [**Table 1**, Affected countries: *r*(154): –0.6085, *P* = <0.0001; All countries (affected + non-affected) *r*(179): –0.4973; *P* = <0.0001]. The consideration of only BCG countries slightly decreases the correlation that could be potentially indicative of the important role of exposure to the *M. tuberculosis* complex in industrialized No-BCG countries with overall higher “hygiene” as well. This view gets support from the existence of negative correlation in No-BCG countries belonging to very high HDI (See the correlation analysis for countries of very high HDI subgroup in **Table 1**). Looking at the distribution of BCNS cancer incidence, it seems to be decreasing exponentially with the increase in TB incidence rates of the countries (*i.e.*, increased probability of natural exposure: priming and boosting). The seeming outlier of the BCG vaccination group that reported BCNS cancer incidence of 5.4 (*i.e*., Montenegro) as well as other high incidence reporting countries happened to be some of the least TB incidence (*M. tuberculosis* complex exposure) countries of the No-BCG and BCG group of countries (**Figure 1B**). The increase in TB incidence rates up to 100 per 100,000 (100k), is seemingly associated with a four-fold reduction in BCNS cancer incidence among 0-4Y-olds, which further goes down by two-fold on an increase in TB incidence to 200 per 100k. The same trend follows till the highest TB incidence rate of about 600 per 100k, potentially indicating TB incidence rates in populations as a risk factor inversely correlated with early childhood BCNS cancer incidence rates.

## Childhood BCNS cancer incidence negatively associated with the natural “boosting” possibility in very high-, high-, and medium- human development index countries

The reporting of childhood BCNS cancer incidence across countries has not been uniform. The projected estimates could be more skewed in low HDI countries because of the possible lack of education, medical infrastructure, diagnostics, affordability, and reporting. Historical estimates for many countries display fluctuations in 3–8-year periods with extreme variations on sex-specific incidence rates, potentially indicative of more imprecise estimates, and underline a need for better data reporting and collation than current (37). Thus, consideration of the latest BCNS cancer incidence estimates in very high HDI (vh-HDI) and high HDI (h-HDI) countries and to a lesser extent medium HDI (m-HDI) may be expected to more reliably illustrate the association, if any. The low HDI countries alone may not reliably show any significant association due to the supposed high *M. tuberculosis* complex exposure (supposedly not a limiting factor any more) and extremely low BCNS incidence rates combined with diagnostic and reporting limitations, including the TB incidence rates. Remarkably, the BCNS cancer incidence among 0-4Y-olds in vh-HDI, h-HDI and m-HDI countries during 2020 was found to be significantly but negatively correlated with their TB incidence rates, except in the low-HDI countries as expected [**Figure 1C** (i-iv); vh-HDI: *r*(65): −0.2990, P = 0.0155; h-HDI: *r*(42): −0.3446; P = 0.0254; m-HDI: *r*(38): −0.3840, P = 0.0173; l-HDI: r(34): 0.0271, P = 0.8789; See **Table 1**]. For the associations in various subgroups, refer to **Table 1**. The negative association between childhood BCNS cancer incidence and exposure to the *M. tuberculosis* complex significantly improved on consideration of only those countries that were reporting incidence of childhood BCNS cancer (affected countries) both as a group of countries (ALL) as well as HDI subgroups [Total: *r*(154): −0.6085; P = <0.0001; vh-HDI: *r*(59): −0.3212, P = 0.0131; h-HDI: *r*(36): −0.5975, P = 0.0001; m-HDI: *r*(29): −0.5468, P = 0.0021]. The negative association remained prominent for relevant subgroups of countries that had significant *M. tuberculosis* complex exposure and BCG vaccination policy in place [BCG countries: ALL affected *r*(125): −0.5595; P = <0.0001; h-HDI: *r*(34): −0.6072; P = 0.0001; m-HDI: *r*(29): −0.5467; P = 0.0021; **Table 1** subgroups]. Together, they suggest that natural boosting combined with BCG vaccination may be protectively related to early childhood BCNS cancer incidence.

## Discussion

The early childhood BCNS cancer incidence in countries appears to be negatively associated with the prevailing possibility of exposure to the *M. tuberculosis* complex. The observations presented could be construed as indicative of non-exposure to *M. tuberculosis* complex or natural “boosting” as an unidentified risk factor for childhood BCNS cancer, possibly functionally related to the “Hygiene Hypothesis” and associated trained immunity. The boosting event(s) could be necessary for supposed priming or activation (immunomodulation), and reprogramming of the metabolism for desired outcomes. We suspect the current regimen of single neonatal BCG inoculation may not be sufficient to cause desired immune activations in environments lacking boosting opportunities. Possibly, multiple shot regimens, as used to be given earlier in many countries (39) or suggested for enhancing nonspecific effects (55, 56) may be necessary for providing the required stimulus and boosting to stem the steady annual rise of early childhood BCNS cancer incidence globally (37). However, since a large majority of early childhood BCNS cancers are supposedly embryonic developmental remnants, the likelihood of the mother’s immune system getting modulated by exposure to BCG and/or *M. tuberculosis* complex itself and in effect modulating and moderating that of the embryo (*in utero*, including endocrine) for ‘normal’ development remains a possibility (20, 28). We would be inclined to suggest the pouring and pooling of resources for a thorough evaluation of BCG’s potential in reducing childhood BCNS cancer incidence. With BCNS cancers in children on the rise, carefully planned, appropriately controlled, multicentric clinical trials, preferably coordinated and managed by WHO, should be performed in high-incidence countries, both No-BCG and BCG countries, to reliably evaluate the qualitative and quantitative contributions of neonatal BCG inoculation(s), boosting, and the mother’s immune system modulation. The countries with zero incidence rates could provide additional insights in terms of etiological agents as well as help identify unknown risk factors specific to countries that are responsible for huge differences in the BCNS cancer incidence rate even among socially similar vh-HDI countries (*e.g*., Iceland, Luxembourg *vs.* Germany, Ireland, Greece, Montenegro, etc.). If immunomodulation has a role in the increased incidence of BCNS cancers in childhood, the recent change in schedule and policy from multiple to single to no BCG inoculations could be costing us >10-fold higher incidence and mortality.

The inherent limitations of the cancer incidence dataset arising from data availability and the methodology employed (57), especially in lower HDI countries, should also be considered when planning for exploratory evaluation studies. As BCNS cancer incidence reporting globally remains fragmentary (37, 57), the studies, whether prospective or registry-based retrospective, should wherever possible be performed in high incidence but low TB prevalence countries, carefully controlling for age, trained immunity status, mothers’ immunization, *etc.* to arrive at meaningful conclusions about its preventive interventional potential. Consideration of existing trends of incidence in previous relevant years (37) as a baseline could provide additional pointers to help reliably estimate the impact of specific interventions in clinical trials.

## Conclusion

BCG vaccinations coupled with timely natural “boosting” could be causally associated with a reduction in childhood BCNS cancer incidence. Future research may be directed at evaluating the same in carefully controlled trials and identifying country-specific differences and modifiers. It is needed to improve our understanding of the role of BCG vaccination and the mechanistic details of the potential protective immune augmentation process. It may help devise ways to stem the steady rise of early childhood cancer incidence with similar components. Additionally, the findings could indicate inoculation regimens that are better suited for lowering early childhood cancer incidence. The revised multiple BCG vaccinations, as they used to be, may have to be reintroduced globally to reduce childhood cancer incidence. The countries with higher incidence rates (ASR per 100k), *e.g*., Montenegro (5.4), Estonia (4.3), USA (4.3), Greece (4.2), Canada (4.1), Israel (4), Slovakia (3.9), Slovenia (3.9), etc., are going to gain the most in terms of morbidity and mortality reduction. However, a lot needs to be done to reliably ascertain the potential benefits of BCG vaccination in reducing cancer incidence, or the lack thereof, before this could be again reintroduced or written off.

## Author contributions

SS conceived the idea, designed the research, analyzed the data, and wrote the paper. RS and AD wrote the paper. All authors contributed to the article and approved the submitted version.

## Supplementary material

Supplementary Figure 1 Estimated Annual Percentage Change of Brain and Central Nervous System Cancer Incidence ASR per 100,000 in 0-4Y-olds. Majority of No-BCG and BCG countries/territories (n=44) display positive annual percentage change in combined incidence for sexes in 0-4Y-olds (last 15 years data). Bars show CI (Available at https://gco.iarc.fr/overtime/en/dataviz/eapc?populations=38000_3600_7600_10000_11200_12400_15200_15600_17000_19100_18800_20300_20800_21800_25000_27600_23300_35200_35600_37200_37600_39200_41000_41400_42800_44000_47000_52800_55400_57800_61600_70300_70500_72400_75200_75600_76400_79200_80000_80400_82630_82610_84000_82620&sexes=1_2&multiple_populations=1&years=2018&cancers=23&types=0&key=asr&age_end=0&group_cancers=0&multiple_cancers=0&mode=population&age_start=0&group_years=0&eapc_span=15&ul=1). The historical estimates remain weak. The observed sex-specific incidence variation in the past for specific years or over time could be indicative of reporting issues. (Available at https://gco.iarc.fr/overtime/en/dataviz/trends?populations=38000&sexes=1_2&multiple_populations=1&years=1943_2018&cancers=23&types=0&key=asr&age_end=0&group_cancers=0&multiple_cancers=0&mode=population&age_start=0&group_years=0).
