# Supplementary figures and images for: BCG vaccination policy, natural boosting and pediatric brain and CNS tumor incidences
Source: Front Immunol. 2023 Jun 13;14:1174006. doi: 10.3389/fimmu.2023.1174006 (PMC10295148; doi:10.3389/fimmu.2023.1174006)

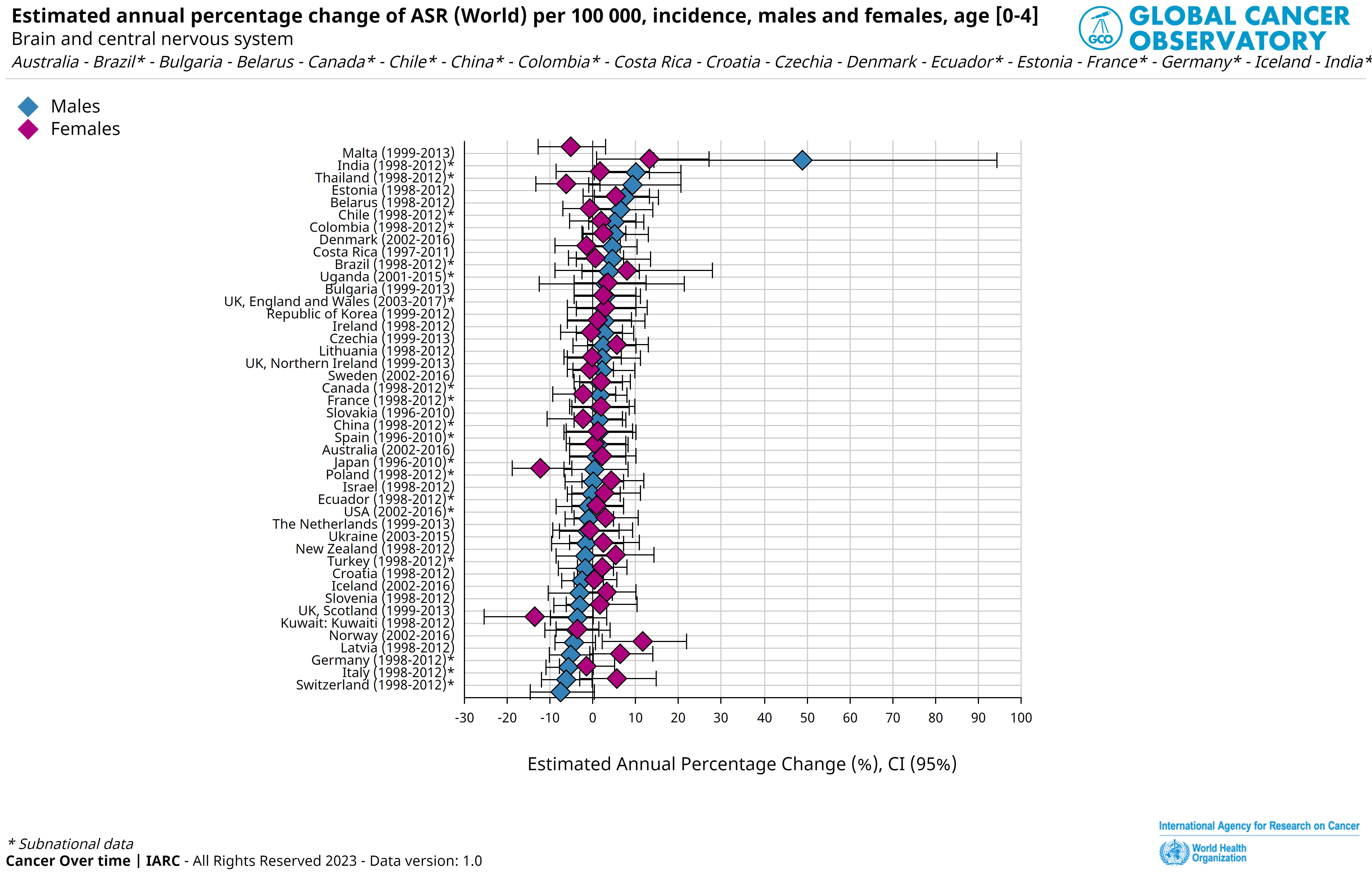

Supplement: Supplementary Figure 1 — Estimated Annual Percentage Change of Brain and Central Nervous System Cancer Incidence ASR per 100,000 in 0-4Y-olds. Majority of No-BCG and BCG countries/territories (n=44) display positive annual percentage change in combined incidence for sexes in 0-4Y-olds (last 15 years data). Bars show CI (Available at https://gco.iarc.fr/overtime/en/dataviz/eapc?populations=38000_3600_7600_10000_11200_12400_15200_15600_17000_19100_18800_20300_20800_21800_25000_27600_23300_35200_35600_37200_37600_39200_41000_41400_42800_44000_47000_52800_55400_57800_61600_70300_70500_72400_75200_75600_76400_79200_80000_80400_82630_82610_84000_82620&sexes=1_2&multiple_populations=1&years=2018&cancers=23&types=0&key=asr&age_end=0&group_cancers=0&multiple_cancers=0&mode=population&age_start=0&group_years=0&eapc_span=15&ul=1). The historical estimates remain weak. The observed sex-specific incidence variation in the past for specific years or over time could be indicative of reporting issues. (Available at https://gco.iarc.fr/overtime/en/dataviz/trends?populations=38000&sexes=1_2&multiple_populations=1&years=1943_2018&cancers=23&types=0&key=asr&age_end=0&group_cancers=0&multiple_cancers=0&mode=population&age_start=0&group_years=0). [file Image_1.jpeg]
